# A New Universal Bundle Theory

**DOI:** 10.1007/s11406-017-9937-6

**Published:** 2017-12-18

**Authors:** Ruoyu Zhang

**Affiliations:** Department of Philosophy, Durham University, Durham, UK

**Keywords:** Bundle theory, Universals, Indiscernibles, Instantiation

## Abstract

Universal Bundle Theory (UBT) holds that objects are fundamentally identical with bundles of universals. Universals are multiply instantiable properties. One popular objection to UBT concerns the possibility of distinct indiscernibles. There are mainly two replies in the literature, corresponding to two representative UBTs, which I shall call the Identity-View and the Instance-View. Each view faces serious problems. This paper proposes a new version of UBT and argues that it is better than these other two versions.

This paper has four sections. Section 1 introduces the Universal Bundle Theory and an objection concerning distinct indiscernibles. Section 2 discusses two common replies (the Identity-View and the Instance-View) to this objection and shows that each of them is problematic. Section 3 proposes a new version of UBT and argues that it is better than the Identity-View and the Instance-View. The relationship between universals and their instances is considered. Section 4 summarises the main conclusions of the paper.

## UBT and the Problem of Distinct Indiscernibles

Universal Bundle Theory provides a one-category ontology. According to this theory, objects are fundamentally identical with bundles of universals. Universals are repeatable qualities and wholly present ‘in’ the objects that instantiate them. For instance, two exactly similar white tables are similar in colour because they literarily share one universal, whiteness.^1^

But consider a universe where there are *only* two symmetrically arrange spheres with the same properties as described by Black (1952).

“*Isn’t it logically possible that the universe should have contained nothing but two exactly similar spheres? We might suppose that each was made of chemically pure iron, had a diameter of one mile, that they had the same temperature, colour, and so on, and that nothing else existed. Then every quality and relational characteristic of the one would also be a property of the other. Now if what I am describing is logically possible, it is not impossible for two things to have all their properties in common*”. (p. 156)

Most people take this thought experiment as a refutation of the commonly known, and attractive Principle of the Identity of Indiscernibles (PII), which holds that, roughly, necessarily for any object x and object y, if x and y share all their properties, x and y are identical.^2^ This in turn suggests that UBT is false, because UBT seems to imply that the spheres are identical.

According to UBT, an object is nothing but its ontological constituents and universals are the only ontological constituents it has. Then suppose the two spheres are named A and B^3^: if sphere A is identical with a bundle of universals U1, U2 and U3 and sphere B is also identical with a bundle of universals U1, U2 and U3, then it seems sphere A must be identical with sphere B. They cannot be distinct because they are constituted by the very same entities. But as demonstrated in the Blackian universe vividly, it seems metaphysically possible for there to be two distinct but indiscernible spheres. This suggests that UBT is false, since it is unclear how it can accommodate that possibility.

## The Instance-View, the Identity-View and their Problems

Despite there being various bundle theories of universals, only two representative versions of UBT tackle the Blackian objection head-on^4^^,^ 5: The Instance-View and the Identity-View. This section evaluates them in the light of recent literature.

### The Instance-View

In his 2004, Rodriguez-Pereyra argues that UBT should hold that an object is identified with an instance of a bundle, instead of the bundle itself. Following Curtis (2014), we call this version of UBT the Instance-View. According to this theory, two objects can instantiate the same bundle of universals because they each correspond to distinct *Bundle-Instances*. Bundles, *like* universals, are capable of being wholly present ‘in’ many instances. If this view is taken, the distinct indiscernibles objection cannot be used against UBT. This is because the Instance-View accommodates the genuine possibility of distinct indiscernibles.^6^

It is easy to *say* there are two Bundle-Instances of the same bundle, but do we have any independent reason to believe that there are distinct entities which share the same constituents? One main reason offered by Rodriguez-Pereyra (2004, p.76) appeals to well-known case of *states of affairs*. For instance, two states of affairs can have the same the constituents. ‘Tom loves Mary’ and ‘Mary loves Tom’ seem to share the same three constituents and yet they constitute distinct states of affairs (Armstrong 1986). If this phenomenon is taken metaphysically seriously, then we have a good reason to reject the Principle of Identity of Constitution (PIC), which is implicitly used in the argument against UBT above.

**PIC: Necessarily, for any objects** ***a*** **and** ***b*** **, if U is a constituent of** ***a*** **if and only if U is a constituent of** ***b*** **, then** ***a*** **is numerically identical with** ***b***

This principle is equivalent to the more familiar Principle of **Extensionality** of parthood, which says two objects cannot have the same parts. Because the notions of part and constituent will play a role throughout our discussion, it may be helpful to clarify them before we discuss the problems of the Instance-View: We will use the word ‘part’ in as broad a sense as possible, and assume all parts are *proper* parts which are not identical with the whole. It is from the Latin verb ‘par’ which just means ‘divide’. Anything ‘divisible’ in some way should be said to have ‘parts’ in the broadest sense. Now we need to distinguish *different ways of having parts*: The composition of Tom, the loving relation, and Mary seems to be different from the composition of three particular lines and the triangle they compose. Only the latter obeys *Extensionality*: three particular lines cannot compose two different triangles. Different philosophers have different terms to mark this distinction. The most famous one might be from Armstrong (1997), when he uses ‘part’ and ‘mereological composition’ for the composition obeying *Extensionality* and reserves the terms ‘constituent’ and ‘non-mereological composition’ for his states of affairs.^7^^,^ 8

Equipped with the distinction made above, let us go back to the Instance-View. Note that Rodriguez-Pereyra himself does not describe Bundle Instances as states of affairs directly. The example of states of affairs just gives him an independent motivation to reject the PIC or Extensionality. But the fact that the PIC is violated by states of affairs does not guarantee it will also be violated by Bundle-Instances. There must be an *analogues* story to be told about *how* two Bundle-Instances can be formed from the same bundle, and what relationship there is between the *Bundle-Instance* and the Bundle. Unfortunately, it is not clear that such an explanation of ‘bundle instantiation’ is provided at all. This might not be a decisive objection but suffice it to say that it would be more parsimonious if the UBT theorist could explain the distinction of the spheres without appealing to the notion of bundle instantiation.

More seriously, most motivated bundle theorists try to give the notion of ‘bundle’ a minimal ontological role and therefore would not find it desirable for the bundling relation to be *reified* as anything *external* to its properties (Simons 1994; Denkel 1997; Morganti 2009). But it seems the Instance-View has to reify bundling relation in order to explain the distinction between a bundle and bundle-instances: If both a bundle and its instances are constituted by the same universals, then why can a bundle be multiply instantiated many times while a Bundle-Instance cannot? As Curtis (2014) asks,

“*Consider a multiply located bundle of universals Bun and one of its instances Ins. Bun has the property of existing at more than one place, but Ins does not. But Bun and Ins are by hypothesis constituted by exactly the same universals. So how can they differ in their properties in this way? What is it that grounds this difference between Bun and Ins?*” (p. 302)

There must be something *else* other than the universals themselves to explain the distinction between bundles and bundle-instances, otherwise the distinction is mysterious: this is the *Grounding Objection*.^9^^,^ 10

We have considered the Instance-View and its problems. Now let us turn to the other main way of defending UBT.

### The Identity-View

The Instance-View holds that objects are identical with Bundle-Instances. By contrast, O-Leary Hawthorne (1995) and Curtis (2014) argue for a different thesis called the ‘Identity-View’: an object instantiates universals by being directly identical with the bundle of them. According to this view,

“*Universals, by definition, are things that can exist wholly and completely at some distance from themselves, and so the defender of the Identity View ought to think that bundles of them can also exist wholly and completely at some distance from themselves too.”* (Curtis 2014, p. 303)

So, according to this Identity-View, the ‘two’ spheres are a single bundle of universals. If an object is identical with the bundle directly and if, as both sides seem to agree, a sphere is an object, then the Identity-View’s interpretation of the Blackian case is very simple: there is only one sphere, a sphere bi-located, or a sphere being miles away from itself. But at any rate, there is only one sphere, instead of two.

The Identity-View is more conservative than the Instance-View in the sense that no extra Bundle-Instances are needed. But the biggest worry is that it simply changes the topic or is ‘modally challenged’ (Rosenkrantz 1994; Zimmerman 1997; Rodriguez-Pereyra 2004). The two-sphere world has two indiscernible spheres, but the Identity theorist’s world only has one. Note that the Identity-View outlined above *entails* that there is only one bundle of universals. Unlike the Instance View, it is *incompatible* with the possibility there being two bundles because there is only one set of universals being bundled, and the sphere must be identical with the bundle rather than the Bundle-Instance, as the name of the view requires. It seems such an incompatibility is enough to make some start to suspect: why cannot there be two spheres or two bundles? Is the one-sphere world and Black’s two -sphere world really the same world? Is the former world qualified to be called the ‘Blackian world’? Consider Zimmerman’s (1997) remark:

“*It is a redescription, is it not? Black’s world contains two spheres. But your bi-located bundle is just one thing that shows up in two places.*” (p.306)

The worry is that the original description and the redescription are not merely two different labels. They clearly seem to express different possibilities and both the possibilities they express appear to be conceivable. Merely *saying* the description given by the Identity View is the *only correct* description of Black’s case is hard to accept. It does not conserve common sense and it reduces the space of possible worlds unnecessarily. This is because the Identity-View cannot accommodate our possibility where there are two spheres. The straightforward fact that we can clearly and easily conceive the two situations as distinct strongly suggests that believing these are different possibilities is intelligible.^11^ No incoherence appears to be involved, and pre-theoretically we have no motivation to deny the two-sphere possibility.

Another general worry about the Identity View is that it seems that it must deal with different Blackian cases *piecemeally*: If there are three spheres in a line and one of them is in the middle, then these ‘three’ spheres will *not* be indiscernible at all: only the one in the middle is “surrounded”, while the two outermost spheres are indiscernible. But then the Identity-View cannot identify those three spheres in one step: it must identify the two outer-most “spheres” to get one sphere, and then identify that with the one in the middle. But then the two outer-most spheres cannot be said to be *bi-located* anymore because the two might be co-located. The three spheres seem to be discernible but the conclusion is that they are not. It is not hard to envisage more complicated cases with four or more numbers of spheres. And in different cases, the Identity-View must apply the identification trick in different stages.^12^

Thus, it seems that the Identity-View does not just deliver counterintuitive results in the two-sphere case. Moreover, it is unclear how the Identity theory can deal with cases involving immaterial objects: if there are two symmetrically arranged *immaterial* entities (like Cartesian minds or angels), it is not clear what analysis the Identity-View could provide because immaterial entities are not spatial at all: expressions like ‘at some distance from itself’ cannot be applied. And insisting in the necessity of spatiality or materialism from the start would be an ad hoc move.

A more conservative and *systematic* view is desirable.^13^ And no matter how the problems immediately above are solved, it should be clear that if a theory can keep the spirit of UBT without denying our intuitive datum about the distinction of Black’s spheres, then that version of UBT should be strongly preferred. Next section is an attempt to provide such a theory.

## A New Bundle Theory of Universals: the Instance Identity-View

### The Instance Identity-View

As we have seen, the Identity View is different from the Instance-View in that only the former identifies objects directly with bundles, rather than Bundle-Instances. However, there are two ways in which a bundle can be metaphysically formed. The bundle could be formed directly from the universals, but it can also be formed from the instances of the universals. Usually when UBT theorists talk about bundles, it is assumed that a bundle is a bundle of universals *simpliciter*, but it seems it is also possible to speak of a bundle of *universal instances*. This corresponds to two sub-versions of the Identity-View, one of which we discussed in section 2.2. Let us now refer to that theory as the ‘Identity Identity-View’, and let us call the new version the ‘Instance Identity-View’. Both views hold that an object is identical with a bundle (not a Bundle-Instance), but one view holds that a bundle is identical with its universals while the other holds that although two bundles can share the same universals, those bundles are not identical with those universals. Rather, the bundles are identical with distinct instances of the universals. Objects are *bundles of instances*, not *instances of bundles*.

**Fig. 1:**
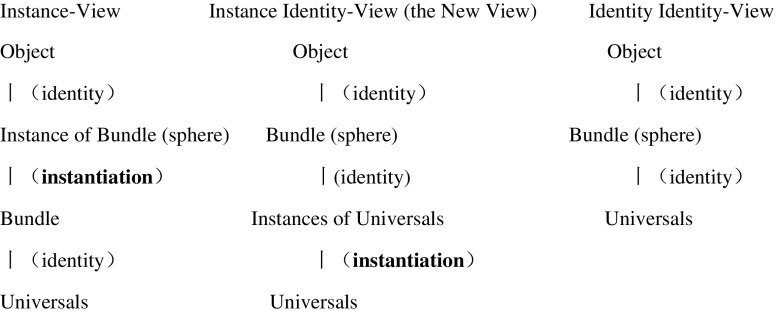
The metaphysical structure of the UBT theories

According to this new view, there can be two spheres in the Blackian case, because each is identical with a distinct bundle of universal instances. The two spheres are indiscernible because both are fundamentally constituted by the same universals, and they are distinct because they are constituted by distinct *universal-instances* and hence identical with distinct bundles (Fig. 1).

On one hand, the New View is different from the Instance-View because firstly, the Instance-View builds the individuality (of the sphere) into the level of instances of the bundle; while on the New View, the individuality of the sphere is parasitic on the individuality of the universal instances. Secondly, the two views differ on the metaphysical priority of bundling and instantiation: speaking metaphorically, the Instance View holds that the universals are firstly bundled and then multiply-instantiated while the New View holds that the universals are firstly multiply instantiated and then the multiply-instantiated universals are bundled.

Speaking less metaphorically, the structures just described represent different relationships of ontological dependence. The Instance-View has it that the bundle is prior to its instances, and the situation is similar to the idea (in the material constitution debate) that a whole is said to be prior to its parts; by contrast, in the New View, universal instances are prior to the bundle they form, and so the situation resembles more the idea that a whole is dependent upon its parts. We will say more about this later.

On the other hand, as we saw, the New View is different from the Identity Identity-View because only the former *allows,* but not *entails*, that there *are two bundles of universal-instances*, and hence *two spheres* in the Blackian case.^14^ By contrast, the traditional Identity Identity-View does not allow this. It simply lacks the extra ontological status of universal-instances in the current sense. All there is for the Identity Identity-View is one bundle of universals, and the universal is bi-located, full stop. It is not the case that we have two universals instances *plus* a bi-located universal.

### Why is the New View Better?

Suppose there are N universals fundamentally in the Blackian case. The comparison between the three views of UBT is shown below:

The New View is best thought of as a kind of Identity-View because it identifies objects with bundles, but it is indeed a balanced view between the Instance-View and the traditional Identity-View. Look at the first row of Table 1: The Instance-View and the Instance Identity-View agree that the number of spheres can be two. This is clearly an advantage over the Identity Identity-View: we do not need to deny the intuitive datum that the possibility of two Blackian spheres is genuine, and therefore we can avoid having to deploy the Identity theorist’s radical redescription strategy. In short, the New View avoids the charge that it is modally challenged.

**Table 1:** A comparison of the three versions of UBT

In the Blackian case	Instance view	Instance identity view	Identity Identity view
How many spheres?	2	2	1
How many bundle instances?	2	0	0
How many bundles?	1	2	1
How many universal instances?	0	2 N	0
How many universals fundamentally?	N	N	N

The second row of Table 1 reveals another way in which the New View is better than the Instance View. Firstly, there are no entities between the objects and the universal instances and so it keeps the intimacy between objects and bundles of instances, which is an advantage over the Instance-View in terms of simplicity; secondly and more importantly, the New View does not face the “Grounding Objection” that we discussed in section 2.1. To recall, the Instance-View developed by Rodriguez-Pereyra does not explain why a bundle and a Bundle-Instance differ in character even though both are fundamentally constituted by the same universals. By contrast, the New View does not postulate the Bundle-Instances and hence does not need to account for the ungrounded distinction between Bundle-Instances and bundles.^15^

So, it is arguable that the New Theory is better than other versions in several respects. Now let us have a closer look at some of the details of the view and anticipate some objections.

### The Relationship Between Universals and Universal-Instances in the New Theory

Any view on properties is incomplete without saying something about instantiation. In this sense, our New Theory is conservative because it does not introduce new theoretical entities like Bundle-Instances. But it does take the distinction between a universal and its instances more metaphysically seriously than the Identity Identity-View. It is therefore important to clarify the nature of instantiation further.

Instantiation in the Identity-View is relatively straightforward and innocent: an object instantiates a universal by *having it as its constituent* or part in the broadest sense. By contrast, as we saw in section 2, the nature of instantiation in Rodriguez-Pereyra’s Instance-View is not clear.^16^

What about our New Theory? A detailed story of instantiation may be more demanding in our New Theory because it not only needs instantiation between the universal-instance and the bundle (object) that contains it as a constituent, but also between a universal and its universal-instance. Here we suggest that the New Theory holds a view about instantiation on which the instances are *ontologically dependent* upon the universals, and not vice versa. Two distinct universal-instances are dependent upon the same universal. We could stop there and take these dependence relations to be primitive. But perhaps we can go further by saying more about the kind of dependence relation that is involved. An interpretation that we shall favour is that the dependence in question is constitutive dependence: *universal instances are constituted solely by the universals they depend on*.

Saying that an instance depends on a universal and that the universal is a constituent of an instance is not completely new. There is nothing incoherent in saying that a universal can be wholly *present in* different instances and be their common constituent. Note that there are different ways to make sense of the notion “in” and not all understandings require universals being *spatially located* (Moreland 2001, p.98). Again, we can be neutral in this issue here.^17^

However, there *is* something unusual about our view that universal instances are constituted by universals even if we grant that a universal can be a constituent in many instances: it also requires the universal to be the *sole* constituent.

In this respect, some might object that the distinction between universals and universal-instances is not as intelligible as the distinction between bundles and Bundle-Instances, because only the latter, and not the former, can be analogues of states of affairs. Even if the Instance-View cannot say that instantiation is the same as that in states of affairs, both the Instance-View and Armstrong’s states of affairs reject the *Extensionality* of parthood. And importantly for current purposes, both the Instance-View and Armstrong’s view on states of affairs assume some version of the Principle *of Supplementation*. Roughly speaking, the most relevant formulation of the principle is this:

**Supplementation: If A is a part of B, then there is a** ***remainder*** **C such that (1) C is a part of B, (2) C is not identical with A, (3) C is not a part of A, and (4) A is not a part of C.** 18

As we saw in section 2, for current purposes we may interpret the word *part* as liberally as possible. This Supplementation Principle precludes the possibility of one thing having only one constituent. Then the worry is clear: saying that a universal-instance is *solely* constituted by a universal is incompatible with the Supplementation Principle.

However, if it does make sense for us to say that something can have only one constituent, then a proponent of the New View may reject this principle. Fortunately, cases violating this principle are not hard to find in the philosophical literature. For instance, Fine’s (1994) famous singleton can be said to have only one constituent. The singleton {Socrates} has Socrates as its sole member and depends for its existence on Socrates. There is nothing incoherent in saying that Socrates is the only constituent or a part of the singleton in the broadest sense. This case can not only be used to show that the Supplementation Principle is questionable, but it also parallels with the case of universals and their instances in various aspects. As Tugby (2016) has pointed out, in both cases, one entity ontologically depends on the other, and therefore the dependent entity is explained by the other. The notion of explanation in play here is ‘constitutive explanation’, which contrasts with ‘causal explanation’ (Salmon 1984).^19^ In fact, Fine himself expresses similar points using terminology like ‘embodiment’ and ‘manifestation’. A *variable object* can have different manifestations or embodiments in different situations. And Fine clearly holds that a property F “is a part of /F/” (Fine 2007, p.162), where the embodiment /F/ can be treated as an instance of F.

The view of sets above is certainly intelligible under realist conceptions of set, but it is not impossible to talk about part-whole relations even if antirealist conceptions are accepted: it does not follow from the fact that Harry Potter is *merely* fictional that Harry Potter has no head as a *part*. Within the realist camp, both the traditional *iterative* conception of sets (namely, a set is formed in a *progressive stages*) and the *structuralist* conception of sets (for instance, Parsons 1997) can admit that members are parts of sets in the broadest sense. Although sometimes a structuralist position holds that the singleton is *not made out* of its sole member, the expression ‘making out of’ is ambiguous because it is still said that singletons get their identity from the *sui generis* ways in which they are *related* to their members, which is sometimes called “combination” (Hossack 2007). There seems no obvious reason why one cannot understand combination in a way that involves parts and wholes in the broadest sense. Meanwhile, the main problem with the iterative conceptions of set lies in the *metaphorical* notion of temporal or progressive stages (Wildman 2013), not the part-whole understanding.^20^ In fact, we do not need to argue for or against a particular view about the nature of sets. It is not clear that those views must be rivals and about the very *same kind* of sets. All we need to do for current purposes is to show that there exists an intelligible case involving sets according to which the supplementation is violated.^21^^,^ 22

Empirical counterexamples can also be given. For instance, Lowe (2013) considers the familiar case of Tibbles the Cat. It seems that the very same Tibbles at time T1 can *survive* with only a head (call it Head) at a later time T2. But Lowe asks,

*“How, indeed, could Tibbles becomes identical with something that was previously not identical with Tibbles, since it was previously only a proper part of Tibbles? I think we should still say that Head is only a proper part of Tibbles*.” (2013, p.136)

Lowe’s aim here is to show that the Supplementation Principle can be rejected. We have good reasons to say that Head and Tibbles are not identical.^23^ But still, Tibbles is constituted by nothing more than Head at T2. The core idea is that a cat and a head cannot be identical because they are essentially different *sorts* of being. Similar cases are not hard to find. Mary can happen to be the sole member of a hiking *club*, or the only one who is the member of parliament, but neither a club nor a parliament is the same kind of being as Mary.

More directly, Bynoe (2011) also shows that some kinds of states of affairs, unlike Armstrong’s, do not obey the Supplementation Principle (in the broadest sense). For example, in the case of the state of affairs *identity is self-identical*, only one constituent is contained. And consider Armstrong’s own ‘victory of particularity’: let x be the state of affairs that *Socrates is a particular*, then we have another state of affairs saying that *x itself is also a particular*. But there is no other constituent except x. Of course, it might be said that ‘being a particular’ or ‘being self-identical’ is not a genuine universal. But as Bynoe (2011, p.8) replies, ‘the ease with which we can produce candidate universals that would realize our problem case strongly suggests that such entities are intelligible’.

If some of the cases above are coherent and intelligible, then the Supplementation Principle should be rejected. Of course, one could try to reject the all the cases above in order to defend the principle, but doing so seems unmotivated. The ubiquitous cases we have in the literature are enough to show that rejecting the Supplementation Principle is not implausible. Note however that our aim is not only to reject the Supplementation Principle and make sense of constitution from one constituent, but also to *allow* (not entail) the one constituent to constitute more than one instance. Fortunately, these cases are also not hard to find. For instance, the singleton of Socrates {Socrates}, and the singleton of that singleton, {{Socrates}} are two distinct entities sharing the same fundamental constituent, Socrates, even if in the latter case Socrates is not a member of {{Socrates}}. Given the mere existence of Socrates, these two distinct entities will automatically be there. Or compare the singleton {Socrates} with the identity state of affairs *Socrates is Socrates*: these two entities are clearly distinct but again, only one constituent is needed. There are also cases without sets. Consider the following two states of affairs: Let ‘*x is F and G*’ be *y*. Then it is possible to have both ‘*y is F*’ and ‘*y is G*’. Again, these two states of affairs are distinct but both depend on only one constituent, *y*.

During this lengthy discussion, we have argued for the intelligibility of treating two universal-instances as being constituted by the one universal they depend upon. If this is coherent, then we have taken an important step towards showing that the Instance Identity-View is a genuine contender in the UBT debate. According to this theory, to repeat, an object is identical with a bundle of universal instances. And a universal instance is dependent upon the universal and constituted by it. Both the *Extensionality* and the *Supplementation* can be rejected in relation to this kind of constitution, and for good reasons. Considered systematically, the prima facie possibility of Black’s universe itself already indicates problems for the Extensionality and the Supplementation Principles. They may be good formal principles in mereology but they are too restrictive when it comes to articulating our metaphysical intuitions about constitution and parthood in the broadest sense.

This New View constitutes a defensible theoretical possibility which is better than both Curtis’ Identity Identity-View and Rodríguez-Pereyra’s Instance View in various respects. However, before concluding, we must briefly consider a final objection to our theory, namely, that the Instance Identity-View is not a genuine Universal Bundle Theory because its robust ontological commitment to universal instances seems to shift the theory closer to the Trope Bundle Theory.

### Universal-Instances are Not Tropes

There are various positions in the literature about the relationship between universals and tropes. Fortunately, we may remain neutral about many of these debates.^24^ For current purposes, it suffices to emphasise that UBT is characterised by the claim that objects are fundamentally constituted by universals, as shown in the Table 1 in section 3.2: all three views try to build objects out of universals. And all agree that one universal can have many instances at the same time, even if the mechanism of instantiation can be different. In contrast to this, *trope* theorists hold that tropes and universals are exclusive, and that tropes are fundamental and simple: it seems that tropes must be simple and have no parts in any realist sense *by definition* (see e.g. Gibb 2012, 2016; Hakkarainen and Keinänen 2017). Moreover, most trope theorists do not think that two tropes can literally share any universal, nor do tropes depend for their existence on universals. Hence, even if universal-instances could be called tropes in some attenuated sense, it should be clear that the New View does not accept most the core theses of Trope Theory. Universal-instances are not simple and they share universals as their fundamental constituents.

## Conclusion

After discussing the main problems facing the Instance-View and the traditional Identity-View, the paper outlined a new bundle theory of universals (the Instance Identity-View), which analyses the Blackian case in a more satisfactory way. On one hand, this new theory does not have the worry of being modally challenged; and on the other hand, it does not need to postulate unexplained Bundle-Instances. The paper also gives an analysis of the relationship between a universal and its instance, according to this new theory.

It should be acknowledged that the overall aim of the paper is relatively modest. We have not argued that the Instance Identity-View is completely problem-free and that it faces no other objections. Further details of the theory will have to be developed and we hope that this paper will to prompt more discussion of this unexplored view. In any case, if some of the claims made above are plausible, the Instance Identity-View is a new theoretical possibility that has some merits, at least for those who are attracted to the Universal Bundle Theory.

## Footnotes

1 UBT does not require that *any* character stands for a universal; all it requires is that all characters are fundamentally grounded by universals.

2 We need not discuss the issue of how precisely to formulate PII because it does not matter for current purposes. Also, there are many other similar cases that have been discussed before and after Black in history of philosophy, but it seems his case is the most representative. Different interpretations of Black’s paper can be given. Some argue that it expresses a thesis about reference or perception, while some others take a more realist reading. No matter which interpretation we take, it is enough for us to say that this case can be a good starting point to discuss the issues on identity and properties with which we are concerned.

3 For current purposes, we can assume that this can be achieved and naming does not break the symmetry of the two spheres.

4 There is another mixed view that we shall not discuss in detail here. For instance, Shiver (2014), following Paul (2002), argues for a mereological bundle theory of universals. He uses a liberal notion of mereology, and argues that there are not only two, but three spheres in Black’s world. One is the unlocated sphere, which is a qualitative overlapping part of the other two located spheres. The two spheres are distinct because of the distinction of their location properties. We do not think this is an attractive view for various reasons. For instance, there are many problems in using location to differentiate the two spheres. One problem is that it cannot be used for many non-located things and co-located things; another problem is it seems that location is dependent upon matter, rather than the reverse. Finally, the distinction of locations itself needs to be explained.

5 The only work I know of which hints towards something like the New-View developed below can be found in a recent paper by Rodriguez-Pereyra (2017), where he modifies his original thought by introducing indiscernible universals, and very briefly mentions the possibility of treating two objects as two bundles with the same universals. But he does not say anything further about *how* this might be achieved. And we do not need the controversial notion of indiscernible universals at any rate. So, we will assume that the identity view and the instance view of UBT are the main contenders in the UBT literature.

6 Note that the instance-view itself does not entail the two spheres are two Bundle-Instances, because it depends on what the original bundle is.

7 Heil (2012) also distinguishes spacetime parts from substantial parts. It is not completely clear whether these distinctions involve the same ontological picture or not. Also, Paul (2002) tries to unify different kinds of parthood, which may reflect our inclination here to use the notion of part as liberally as possible. But we need not get into these complexities here.

8 Sometimes, a non-mereological part is taken to be the same as ‘metaphysical part’ (Koon and Pickavance 2015, p.124). This is misleading. It is true that in his debates with Lewis, Armstrong is more ontologically serious about properties and constituent ontology, but some theories might have constituents which qualify as metaphysical parts without rejecting Extensionality. For instance, tropes can be a metaphysical part of an entity but it is not clear whether tropes can compose states of affairs in Armstrong’s sense. At least, if tropes are accepted, then the main argument for states of affairs will be redundant. So we will not use the term metaphysical parts.

9 Note that states of affairs do not face this objection because the constituents are not more fundamental than the states of affairs they constitute. But the Grounding Objection still works for those who understand the Instance-View in terms of material constitution, rather than states of affairs: for example, how can the same stuff constitute both a lump of clay and a statue?

10 There are other related problems we cannot cover here. The Instance-View seems unmotivated in two ways. Firstly, it is unattractive for those who hope to defend the Principle of Identity of the Indiscernibles (PII) because it assumes the sphere case is a counter-example of PII. Secondly, there may be cases in which objects only have one property. This property could be complicated in predication but simple ontologically, like a structural universal or a distributed property. This possibility is not the main concern of this paper but if it is indeed possible that there is only one property involved, then appealing to Armstrong’s states of affairs cannot work because Armstrong’s states of affairs usually contain more than one universal.

11 One further argument in support of this conclusion, which is beyond the scope of the paper, is the Continuity Argument firstly proposed by Adams (1979). Roughly, if it is possible to have two *almost*-indiscernible spheres, and the small difference between the spheres can be modified continuously, then accepting the identity view will force us to accept some unnatural ‘collapse’ from two to one. See Rodriguez-Pereyra (2017) for a recent discussion and reply. Also, there might be a worry about how to apply the identify view because it requires the distinction between symmetrical cases and the non-symmetrical cases. But whether a case is symmetrically arranged or not cannot be easily decided. An apple and a banana might be viewed as symmetrically arranged *under certain description*: suppose for a bundle theorist, an apple is composed of AA and a banana is composed of BBCC, then the situation may be redescribed as two symmetrical ABCs, but should the Identity View identify an apple and a banana under that description? We leave this for readers to consider.

12 It might be replied that those different steps are just steps in *reasoning* rather than stages of some metaphysical mechanism in reality. However, even if this is the case, this still shows that the view is not *ideologically parsimonious*. Moreover, there is a strong indeterminacy in how to apply the various identification steps when reasoning about cases: suppose there are four spheres abcd arranged symmetrically, should we identify ab, and then cd, or ac, and then bd? We think all these considerations are enough to make the identity-view seem arbitrary.

13 There are other problems facing the Identity-View, which challenges its own coherence. For instance, see the debate between Zimmerman (1997), Curtis (2014), Varzi and Calosi (2016), on how to use counterpart theory to explain the possibility of one bundle being two. This issue goes beyond the scope of this paper.

14 There is not entailment because it is conceptually possible to have one instance which can be multiply-located. This is not the same as saying that an instance can be instantiated many times, which violates the meaning of instance, and hence it is conceptually possible that the ‘two’ spheres are only one multiply-located bundle of universal-instances. Location and instance are related but distinct notions.

15 Also, note that it would not be correct to argue that the New-View faces a similar Grounding Objection: the idea that both universals and their instances are constituted by the universals and hence their distinction is also ungrounded. On one hand, it is fine to say bundles are constituted by universals but it sounds odd to say universals themselves are constituted by universals. On the other hand, all parties in the debate share the assumption that it is of the very nature of universals that they can be multiply located at a time. If there is something unexplained at all, then this is a feature of all theories with universals. In contrast, the Grounding Objection is uniquely pressing for the Instance-View because it not only makes the common assumption that universals can be multiply located, but also says the same about the bundles that are instanced.

16 It is worth noting that, for some philosophers, given that universals are ontologically prior to their instances, the characterization of UBT above might sound like a way to *Platonism*. One way to distinguish Platonism from Aristotelianism concerns what Armstrong calls the Aristotelian Principle of Instantiation, which says that universals are *generically dependent* upon their instances, rather than vice versa. For Aristotelians like Armstrong, there cannot be uninstantiated universals. However, it is possible for a bundle theorist to coherently hold that a universal must be instantiated in at least one bundle. This does not mean the bundle is not dependent upon the universals. We will ultimately remain neutral on this issue, however.

17 One worry with identifying instantiation with spatial location, when doing constituent ontology, is that it may be a category-mistake to empirically locate these constituents and ask where these constituents are, because locations themselves should also be subject to the process of world making, being constituents.

18 This is usually called Weak Supplementation in contrast with the strong supplementation. See Simons (1987) for further details.

19 The only dissimilarity might be that the relation between the universal-instances and universals seems to be contingent, in the sense that the universal could exist without particular instances. In contrast, it is necessary that the singleton exists if its member does. However, it is not outlandish to say that universals and their instances are necessarily connected, following later Armstrong or a counterpart theory about universals. But this is not an issue we need to explore here. Likewise, we are open about whether all ontological dependences involve grounding.

20 Wildman (2013) also argues that the *dependence intuition* that Fine relies on is redundant for certain Platonic iterative views about sets, but that does not show it is incoherent. The dependence intuition does not need to assume any theory about sets, rather it is a natural description of how we understand things: we do not need to know what a set is in order to know the nature of Socrates. Even if dependence is given up, what we have ontologically will not be changed. In fact, the picture we presented in section 3.1 could be merely viewed as a graph with lines and nodes under a structuralist view.

21 The only worry that one might have here is that, even if the singleton only has one member, it may still have other formal features which can be part of the singleton. For example, the “bracket” must have some formal ontological status. But as Fine (2007) argues, this does not lead to the intended result because we can always construct higher-level singletons out of those formal features and it makes no sense to talk about formal features of formal features. Hence, the Supplementation Principle is still violated.

22 If we still find the nature of sets mysterious, then consider the idea from what Varzi (1996) calls the “boundary free tradition” of Whitehead and Clarke: intuitively, boundaries are parasitic or secondary entities lacking their own existence. In his theory of extensive connection, Whitehead holds that the open interior of a topologically closed region is a proper part of the region even if there is nothing extra that we can call ‘boundary’.

23 According to Lowe, a cat’s head is just not the right sort of thing to be called a cat. For example, a cat usually has the property of having four legs when it was born but clearly a head does not have that property at any time.

24 As shown in Lowe (2006), it is coherent to include both tropes (modes) and universals in one’s ontological categories.
